# Hydration–Strength–Workability–Durability of Binary, Ternary, and Quaternary Composite Pastes

**DOI:** 10.3390/ma15010204

**Published:** 2021-12-28

**Authors:** Yi Han, Seokhoon Oh, Xiao-Yong Wang, Run-Sheng Lin

**Affiliations:** 1 Department of Integrated Energy and Infra System, Kangwon National University, Chuncheon-si 24341, Korea; hanyii@kangwon.ac.kr (Y.H.); gimul@kangwon.ac.kr (S.O.); 2 Department of Architectural Engineering, Kangwon National University, Chuncheon-si 24341, Korea

**Keywords:** quaternary binder, limestone powder, metakaolin, fly ash, sustainability

## Abstract

At present, reducing carbon emissions is an urgent problem that needs to be solved in the cement industry. This study used three mineral admixtures materials: limestone powder (0–10%), metakaolin (0–15%), and fly ash (0–30%). Binary, ternary, and quaternary pastes were prepared, and the specimens’ workability, compressive strength, ultrasonic pulse speed, surface resistivity, and the heat of hydration were studied; X-ray diffraction and attenuated total reflection Fourier transform infrared tests were conducted. In addition, the influence of supplementary cementitious materials on the compressive strength and durability of the blended paste and the sustainable development of the quaternary-blended paste was analyzed. The experimental results are summarized as follows: (1) metakaolin can reduce the workability of cement paste; (2) the addition of alternative materials can promote cement hydration and help improve long-term compressive strength; (3) surface resistivity tests show that adding alternative materials can increase the value of surface resistivity; (4) the quaternary-blended paste can greatly reduce the accumulated heat of hydration; (5) increasing the amount of supplementary cementitious materials can effectively reduce carbon emissions compared with pure cement paste. In summary, the quaternary-blended paste has great advantages in terms of durability and sustainability and has good development prospects.

## 1. Introduction

Human-made carbon dioxide emissions are an important cause of global climate change, and cement production accounts for 5–8% of human-made carbon dioxide emissions [1,2,3]. Moreover, with the development of industry and infrastructure, the demand for cement will increase substantially; therefore, carbon dioxide emissions will continue to grow predictably [4,5]. At present, reducing cement carbon dioxide emissions has become an urgent problem that must be solved. In recent decades, many researchers have also devoted themselves to research solutions. Supplementary cementitious materials (SCMs) application, recycled concrete, carbon capture, utilization, and storage are common technical solutions to reduce carbon dioxide emissions [6]. Among them, using SCMs to replace cement can reduce carbon dioxide emissions by 30 and 40% and is currently the most economical and effective method [7,8]. In addition, the use of some industrial by-products can also improve the problem of environmental dust pollution.

At present, there are many studies on materials that are used as SCMs to replace cement, such as blast furnace slag, fly ash (FA), silica fume, metakaolin (MK), and natural pozzolan [9,10]. As SCMs, they must have pozzolanic reactivity; they can react with calcium hydroxide and water to produce reaction products such as calcium silicate hydrate (C–S–H), calcium aluminate silicate hydrate (C–A–S–H), or calcium sulfoaluminate hydrate. In addition, many common mineral materials have been widely used in concrete such as limestone (LS) powder and quartz powder. Studies have shown that LS powder and aluminum-rich auxiliary cementitious materials can produce a synergistic effect [11].

In recent years, the development of binary or ternary composite concrete mixed with LS and aluminum-rich SCMs has become a concern. De Weerdt et al. [12] confirmed that LS powder and FA have a synergistic effect when mixed as alternative materials and can improve the compressive strength in a specific ratio. Thongsanitgarn et al. [13] found that when LS powder and FA are used as alternative materials, the compressive strength of ternary concrete is higher than that of ordinary Portland cement (OPC)-FA binary concrete. Du and Pang [14] studied LS powder and MK as alternative materials to prepare high-performance concrete and found that the early compressive strength of ternary- concrete with LS powder and MK was lower than that of pure cement concrete, and the compressive strength increased faster. Dhandapani et al. [15] found that limestone calcined clay cement (LC^3^) concrete exhibits compressive strength comparable to pure cement concrete in the early stage, and with the increase in age, it becomes higher than pure cement concrete in the later stage. In terms of durability, LC^3^ concrete has better resistance to the entry of chloride ions.

In addition, to reduce carbon dioxide emissions, many researchers have studied the reuse of waste products. Jin et al. [16] produced asphalt using waste cathode-ray-tube (CRT) glass powder and found the optimal addition content of CRT glass powders could be up to 10%. Cai et al. [17] prepared autoclaved aerated concrete (AAC) using waste red gypsum to substitute for fly ash. The results showed that the addition of waste red gypsum effectively reduces the gas foaming rate and can increase the compressive strength of AAC when added in an appropriate amount. Yang et al. [18] studied the thermal expansion coefficient of three types of recycled aggregates to prepare concrete. All three types of recycled aggregates reduced the thermal expansion coefficient of concrete. In addition, when the replacement rate of recycled aggregates was the same, the thermal expansion coefficient of the concrete prepared by the equivalent mortar volume method was lower than that of the concrete designed in the conventional way. Lehner et al. [19] analyzed the change of the chloride diffusion coefficient over time based on 32 different binary and ternary concrete mixtures and proposed models based on the coefficient of variation (constant or time-dependent).

Currently, there have been extensive and detailed studies on binary and ternary composite concretes with LS powder, MK, and FA as alternative materials. Still, research on quaternary-blended paste has not been carried out. First, when LS, FA, and MK are used as alternative materials simultaneously, there is a lack of understanding of the workability. Second, there is a lack of knowledge of the early compressive strength and hydration performance of OPC–LS–MK–FA quaternary-blended paste. Third, the durability and sustainable development of OPC–LS–MK–FA quaternary-blended paste also need to be further explored. Fourth, there is a lack of understanding of the hydration products of OPC–LS–MK–FA quaternary-blended paste.

The gaps this study bridged compared to currently existing work are summarized as follows: (1) we compared the workability of binary-, ternary-, and quaternary-blended pastes using LS powder, MK, and FA as alternative materials; (2) we studied the early compressive strength and hydration performance of the OPC–LS–MK–FA quaternary-blended paste, and the correlation between the compressive strength and the heat of hydration; (3) we studied the durability of the OPC–LS–MK–FA quaternary-blended pastes; (4) we conducted X-ray diffraction (XRD) and attenuated total reflection Fourier transform infrared (ATR-FTIR) tests to study the microstructure of the quaternary-blended paste. In addition, the CO_2_ emissions of the blended pastes were calculated, and the strength normalized CO_2_ emissions were determined. The sustainability performance of the OPC–LS–MK–FA quaternary-blended paste also showed satisfactory results.

## 2. Materials and Methods

### 2.1. Materials Characterization

**Figure 1:**
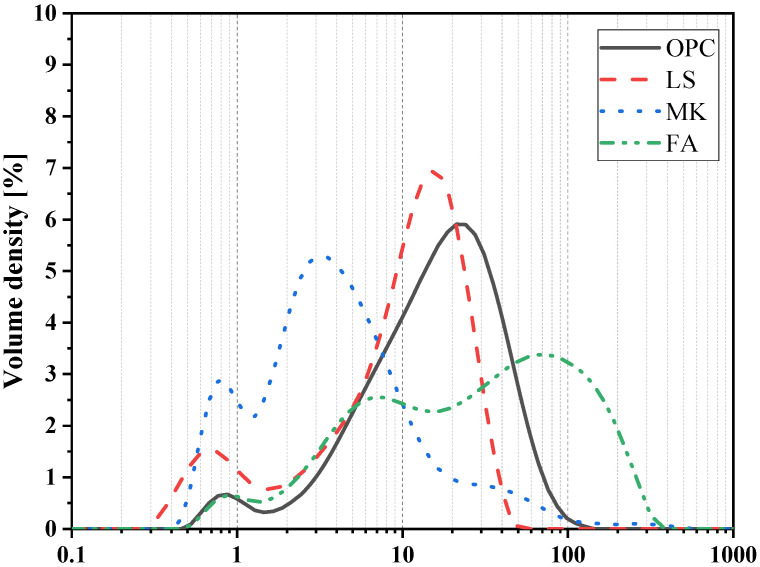
Particle size distributions of binders.

In this study, LS, MK, and FA powder were used as alternative materials for OPC. Table 1 shows the chemical composition and ignition loss of OPC and alternative materials determined by X-ray fluorescence (XRF) analysis. The materials powder was dispersed in alcohol, and the particle size distribution curve of OPC and alternative materials obtained by particle size distribution analysis is shown in Figure 1. We found that the metakaolin was much finer than other components, while fly ash was much coarser than other components. Moreover, the sequence of the average size of binder components was metakaolin < limestone powder < Portland cement < Fly ash. In addition, the d50 and specific gravity of OPC, LS, MK, and FA are given in Table 2.

### 2.2. Mixed Design and Preparation

In this study, all pastes were prepared with w/b = 0.5 according to the ASTM C305-20 [20] standard. A Hobart-type mixer was used to prepare the paste. After preparation, 50 × 50 × 50 mm cube specimens were sealed with a film and stored in a curing chamber at a temperature of 20 ± 2 °C for constant temperature curing, and 40 × 40 × 160 mm cuboid specimens were placed to cure in a 20 ± 2 °C stable temperature water tank.

This study used three alternative materials, namely, LS powder (0–10%), MK (0–15%), and FA (0–30%). The experimental mixing ratio is given in Table 3. Three different blended paste systems, namely, the system with 5% LS, the system with 7.5% MK, and the system with 15% FA, were prepared. Each system contained a binary-, two ternary-, and a quaternary-blended paste. In this way, the differences between the binary, ternary, and quaternary specimens could be discovered. In addition, a pure cement paste (C1-C100) was prepared as a control specimen.

### 2.3. Experimental Methods

#### 2.3.1. Workability Test

In this experimental work, a mini-slump cone was used for the workability test. The mini-slump was 50 mm high, 70 mm diameter at the top, and 100 mm at the bottom [21]. The mini-slump was placed in the center of the steel plate. After the fresh paste was prepared, it was poured into the mini-slump, then a shovel was used to smooth the top. The mini-slump was then lifted vertically, two cross diameters of the paste were measured post-collapse, and the average value was taken.

#### 2.3.2. Heat of Hydration

This study used a TAM-Air (TA Instruments, New Castle, DE, USA) isothermal calorimeter to test the heat of hydration. Because the specific heat capacity of glass is similar to that of cement, glass was used as reference material [22]. First, we set the test temperature of the isothermal calorimeter to 20 °C; after the paste was mechanically mixed, 5 g was quickly put in an ampoule and promptly put into the isothermal calorimeter. The experimental test time was set to 7 days, and the laboratory temperature was kept at 20 ± 1 °C to ensure the accuracy of the experiment [23].

#### 2.3.3. Ultrasonic Pulse Velocity

Ultrasonic pulse velocity (UPV) testing is one of the most commonly used non-destructive testing (NDT) methods. The ultrasonic pulse velocity tester used a portable non-destructive digital indicator tester (Pundit Lab) produced by the Swiss Proceq company (Zurich, Switzerland). It was tested on days 3, 7, and 28 in accordance with ASTM C597-09 [24] standard. The mold size of the paste specimen was 50 × 50 × 50 mm. Three samples of each type of paste were tested. Two tests were performed on each of the four sides perpendicular to the direction in which the paste was poured, and the average value was taken as the final result.

#### 2.3.4. Compressive Strength

The compressive strength test in this study used a cube with a specimen size of 50 × 50 × 50 mm. The compressive strength test was performed according to the ASTM C349-18 [25] standard. The test was performed at the curing times of 3, 7, and 28 days. Three specimens were tested for each mix ratio, and the final result was the average of the results of the three specimens.

#### 2.3.5. Surface Resistivity

As a non-destructive, simple, and fast detection method, the surface resistivity test has been used to evaluate the corrosion resistance of concrete [26]. According to the AASHTO T 358 [27] standard, this article used the ResiPod portable non-destructive resistivity tester produced by the Swiss Proceq company for surface resistivity testing. The prepared 40 × 40 × 160 mm cuboid specimens were taken out of the constant temperature water tank after curing for 3, 7, and 28 days. Each sample was tested on four vertical planes perpendicular to each other, and the average value was taken.

Ages of 3, 7, and 28 days were chosen for non-destructive testing such as UPV and electrical resistivity. Ages 3 and 7 days belong to an early age, and 28 days belongs to a late age. Based on comparisons of the results of UPV and electrical resistivity at different ages, the effect of mineral admixtures on UPV and electrical resistivity of young concrete and hardened concrete could be clarified.

#### 2.3.6. XRD and FTIR

The X’Pert PRO MPD diffractometer (Panalytical, Almelo, The Netherlands) was used to perform X-ray powder diffraction analysis on the specimens at the curing age of 28 days. The sample was scanned from 5° to 70° (2θ) in increments of 0.013°, and the cumulative time of each step was 8.67 s [28].

The FTIR experimental test was carried out using a Frontier spectrometer (PerkinElmer, Waltham, Massachusetts, USA). Transmission mode was used to collect spectra with a resolution of 0.4 cm^−1^. Each sample was scanned 32 times in the range of 500 to 4000 cm^−1^. The background of the ZnSe diamond crystal was scanned before each measurement [29].

## 3. Results

### 3.1. Workability Test

**Figure 2:**
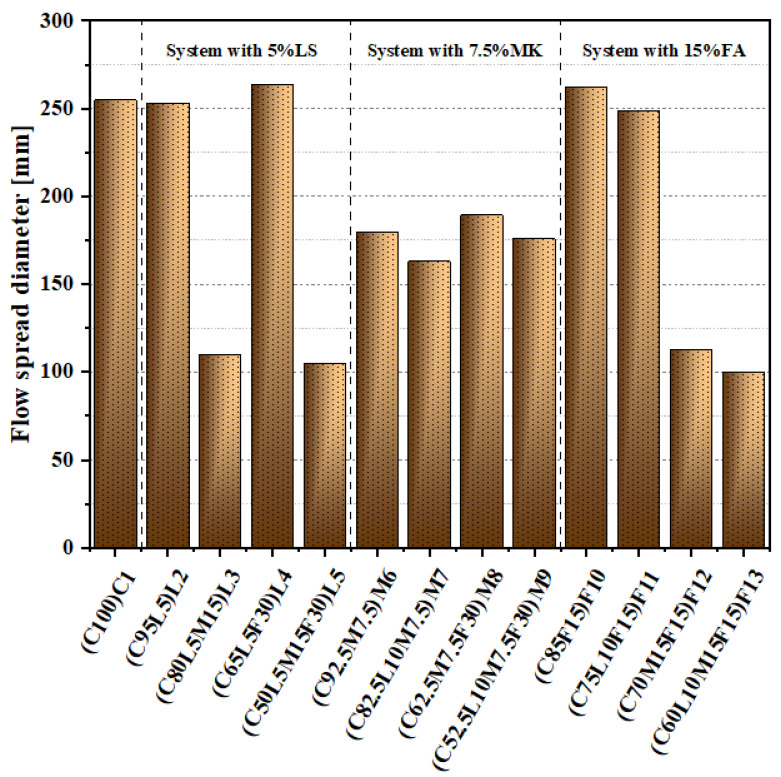
Flow spread values of the blended pastes.

Figure 2 shows the test results of the flow expansion diameter of paste. It can be observed in Figure 2 that the flow expansion diameter of all pastes was divided into three levels. Capital letters C, L, F, M mean cement, limestone, fly ash, and metakaolin, respectively. The first level: C1(C100), L2(C95L5), L4(C65L5F30), F10(C85F15), and F11(C75L10F15) flow expansion diameters were more than 245 mm; the second level: M6(C92.5M7.5), M7(C82.5L10M7.5), M8(C62.5M7.5F30), M9(C52.5L10M7.5F30) flow diameter between 163 and 189.5 mm; the third level: L3(C80L5M15), L5(C50L5M15F30), F12(C70M15F15), and F13(C60L10M15F15) flow diameters were less than 130 mm, and the quaternary-blended paste L5(C50L5M15F30) and F13(C60L10M15F15) had almost no fluidity. The reason for this phenomenon was the amount of MK added. The substitution amounts of the blended paste MK in the three levels were 0, 7.5, and 15%, respectively. This demonstrated that the blended paste’s workability performance decreased with the increase in the amount of MK in the mixture. According to previous studies, the decrease in workability caused by the addition of MK is because of the high Al_2_O_3_ content and powder granular high specific surface area of MK [30].

In the first level, L2(C95L5), with 5% LS added, had only a slight decrease compared with the control specimen C1(C100). Compared with F10(C85F15) and F11(C75L10F15), after adding 10% LS to F11(C75L10F15), the flow diffusion diameter was reduced by 13.5 mm. The same phenomenon was found in the second and third levels. In the second level, compared with M6(C92.5M7.5) and M8(C62.5M7.5F30), the flow expansion diameters of M7(C82.5L10M7.5) and M9(C52.5L10M7.5F30), after addition of 10% LS, were reduced by 27 and 23.5 mm, respectively. In the third level, the flow diameter of F13(C60L10M15F15) was also significantly lower than that of F12(C70M15F15). This demonstrated that adding LS was not conducive to the workability of the blended paste. Jiang et al. [31] also obtained similar results in previous studies. We also observed that FA had a positive effect on performance. For example, compared to the control specimen, for F10(C85F15), the flow expansion diameter increased by 7 mm; in the second level, compared with M6(C92.5M7.5) and M7(C82.5L10M7.5), the flow expansion diameters of M8(C62.5M7.5F30) and M9(C52.5L10M7.5F30), with 30% more FA, increased by 9.5 and 13 mm, respectively. The increased workability of the blended paste with FA can be attributed to the spherical shape of the FA particles and the “ball-bearing effect” that occurred at the contact points between the spherical particles [32].

### 3.2. Heat of Hydration

**Figure 3:**
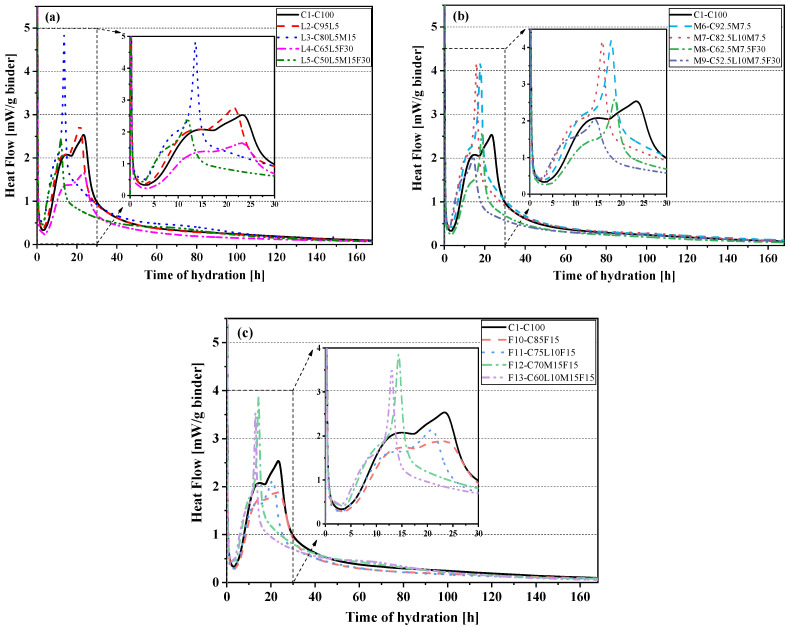
Rates of hydration heat: (**a**) the control specimen and LS system; (**b**) the control specimen and MK system; (**c**) the control specimen and FA system.

The seven-day hydration heat curve of the paste in this paper is shown in Figure 3. Figure 3a–c shows the hydration heat release curves of the control specimen (C1-C100) and the LS system, MK system, and FA system. The addition of alternative materials changed the hydration kinetics of the paste. The hydration heat release curve of the blended paste with different substitution amounts was changed to different degrees than the pure cement paste (C1-C100). Generally, the hydration process of cement can be divided into five periods: the initial period, the induction period, the acceleration period, the deceleration period, and the slow development period [33,34]. The induction period of the paste can be observed in Figure 3a–c; it was found that the heat release of L2(C95L5) and M6(C92.5M7.5) in the induction stage was higher than that of the control specimen C1(C100), but the F10(C85F15) blended paste was lower than C1(C100). This showed that LS and MK increased the heat release during the induction phase, while FA reduced the heat release. Moreover, compared with C1(C100), the L2(C95L5) blended paste induction phase ended (the acceleration phase appeared) earlier, but the M6(C92.5M7.5) and F10(C85F15) blended paste had no change.

We observed that there were two reaction peaks in the main hydration peak of the hydration calorimetry curve. The first reaction peak was caused by C3S hydration; the second reaction peak was from the aluminate phase reaction [35]. In Figure 3a, compared with the pure cement paste (C1-C100), the time of appearance of the silicate reaction peak and aluminate exothermic peak of the L2(C95L5) blended paste containing 5% LS was approximately 1.7 h earlier. This may be because the presence of LS increased the nucleation sites of the hydration product, thereby promoting the early hydration reaction of the L2(C95L5) blended paste [27]. In Figure 3b, the time of appearance of the aluminate exothermic peak of the M7(C82.5L10M7.5) and M9(C52.5L10M7.5F30) blended pastes was also significantly earlier than that of the M6(C92.5M7.5) and M8(C62.5M7.5F30) blended pastes. This may also be because the M7(C82.5L10M7.5) and M9(C52.5L10M7.5F30) blended pastes replaced 10% more LS than the M6(C92.5M7.5) and M8(C62.5M7.5F30) blended pastes. Additionally, in Figure 3c, the time of appearance of the aluminate peaks of the F11(C75L10F15) and F13(C60L10M15F15) blended pastes with 10% LS added was also advanced to different degrees. The blended paste’s aluminate peak with 15% MK (such as L3-C80L5M15, L5-C50L5M15F30, F12-C70M15F15, and F13-C60L10M15F15) became sharper. The aluminate exothermic peak time was also significantly earlier, and the aluminate exothermic peak was found; it merged with the silicate reaction peak. Figure 3b shows the MK blended paste system. It was found that compared with pure cement paste (C1-C100), the heat flow curve of the blended paste of the MK system had different degrees of leftward displacement, which also showed that MK accelerated the hydration reaction rate of the blended paste. Vance et al. [36] also reported similar results. Figure 3c shows that the two reaction peaks of the F10(C85F15) blended paste with 15% FA added were significantly reduced. In Figure 3a,b, the peak value of the blended paste’s aluminate reaction with 30% FA added had a similar decrease. For example, the quaternary-blended paste L5(C50L5M15F30) had a lower aluminate peak value than the L3(C80L5M15) blended paste, which decreased by 49.7%. This was previously reported in reference [37]. It is also worth noting that the blended pastes L3(C80L5M15), L5(C50L5M15F30), and F13(C60L10M15F15) showed a reaction peak at about 60 h; this was due to the production of hemicarboaluminate (Hc) and monocarboaluminate (Mc) [38]. In addition, M7(C82.5L10M7.5) and M9(C52.5L10M7.5F30) showed a reaction peak at about 90 h, and the peak was weaker. This meant the position and intensity of this peak were affected by the amounts of LS and MK added. As the aluminate contents in the binder increased, the intensity of peak was enhanced, and the peak occurred earlier.

**Figure 4:**
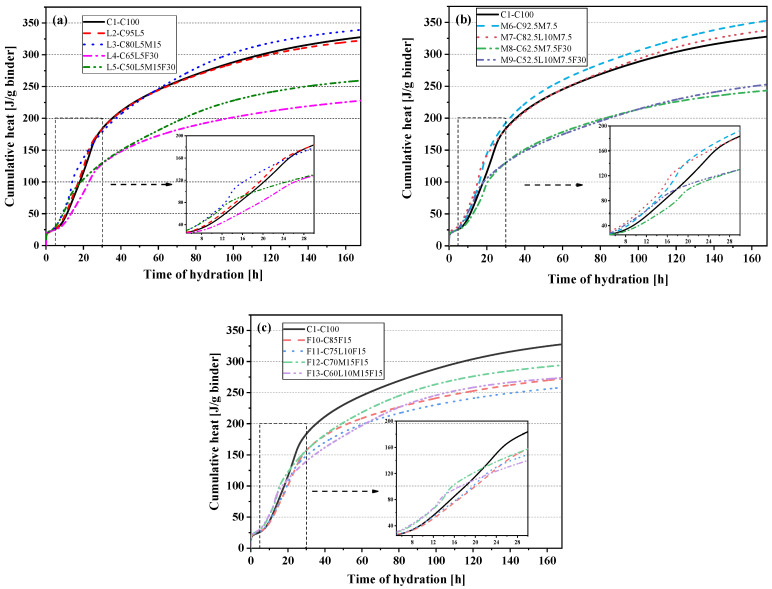
Cumulative heat of hydration: (**a**) the control specimen and LS system; (**b**) the control specimen and MK system; (**c**) the control specimen and FA system.

Figure 4a–c show the cumulative calorimetry curves of the LS system blended paste, MK system blended paste, and FA system blended paste, respectively. The accumulated heat of the M6(C92.5M7.5) blended paste was the highest, reaching 352.66 J/g. The quaternary mixtures all showed low cumulative heat release. In the LS system mixture shown in Figure 4a, the heat release rates of the L2(C95L5), L3(C80L5M15), and L5(C50L5M15F30) blended pastes in the first 17 h all exceeded the pure cement paste (C1-C100), and the 7 days cumulative heat release of the L3(C80L5M15) blended paste was 14.47 J/g higher than the C1(C100) paste. The cumulative heat release curve of the L3(C80L5M15) blended paste increased significantly at 14 h from the aluminate reaction. At approximately 60 h, the cumulative heat release of L3(C80L5M15) further increased from the Hc and Mc [39]. The 7-day cumulative heating value of L5(C50L5M15F30) was 251.41 J/g, 23.28% lower than that of pure cement paste C1(C100). As shown in Figure 4b,c, the accumulated heat of the M6(C92.5M7.5) and M7(C82.5L10M7.5) blended pastes of the MK system exceeded that of the pure cement paste (C1-C100) in 7 days. Compared with the C1(C100) paste, the calorific value of the FA system blended paste was reduced to different degrees in 7 days. The slope of the cumulative heat release curves of the quaternary-blended pastes L5(C50L5M15F30) and F13(C60L10M15F15) slowed down at about 12 h, and the cumulative heat release increased rapidly at 60 h. This increase may be due to the increased heat release caused by the production of Hc and Mc [38]. This also confirmed the results observed in Figure 3.

**Figure 5:**
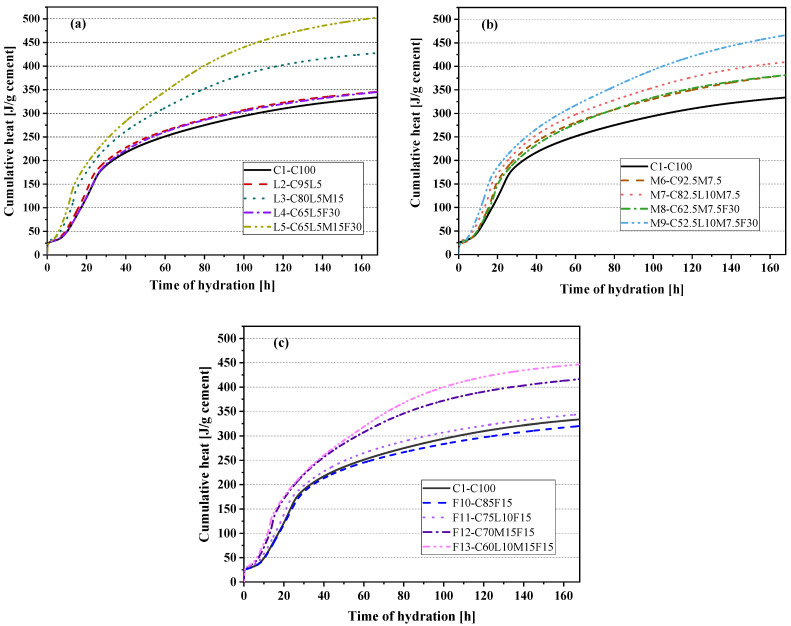
Cumulative hydration heat normalized to cement content: (**a**) the control specimen and LS system; (**b**) the control specimen and MK system; (**c**) the control specimen and FA system.

Figure 5 shows the cumulative hydration heat normalized to cement content. It can be seen that the cumulative heat of hydration of the OPC–LS–MK–FA quaternary paste in each system was the highest. This indicated that the addition of LS–MK–FA helped cement hydration, mainly because of the dilution effect produced by the alternative materials. It is worth noting that only F10(C85F15) of all the mixed pastes had a lower cumulative heat of hydration than the control group C1(C100). This was because fly ash delayed the effect of cement hydration.

### 3.3. Compressive Strength

**Figure 6:**
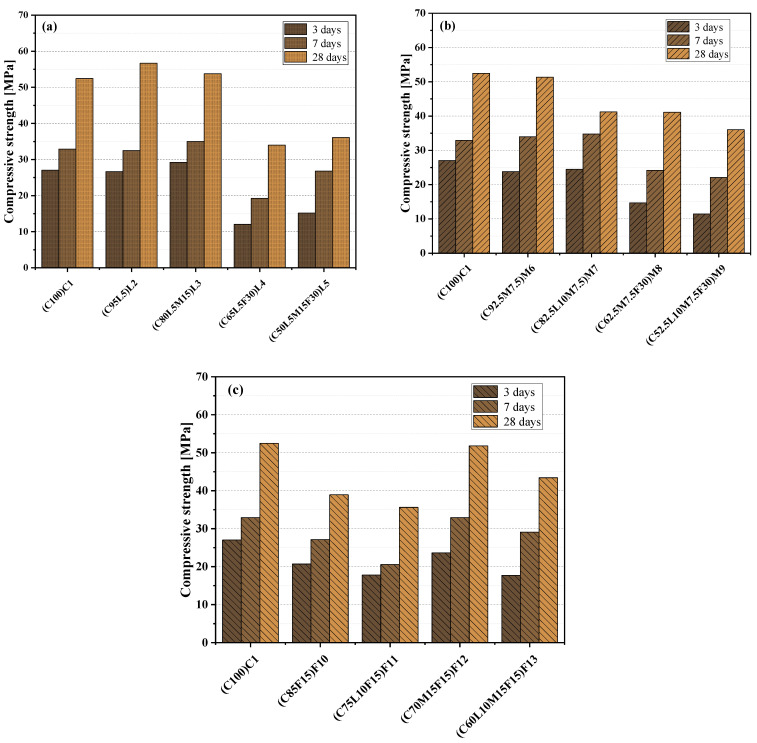
Compressive strength: (**a**) the control specimen and LS system; (**b**) the control specimen and MK system; (**c**) the control specimen and FA system.

Figure 6 shows the change in compressive strength of the paste at 3, 7, and 28 days. Generally, LS, as an alternative material added to the blended paste, had a diluting effect and a nucleation effect [40]. Therefore, it is expected that in the early stage of hydration, LS can provide the compressive strength of the blended paste. As shown in Figure 6a, the compressive strength of L2(C95L5) blended paste with 5% LS added in the early stage (i.e., 3, 7 days) was equivalent to that of pure cement paste; when the curing age reached 28 days, the compressive strength of mixed L2(C95L5) exceeded that of C1(C100), reaching 56.59 MPa. In Figure 6b, the blended paste M6(C92.5M7.5) with 7.5% MK added exhibited compressive strength equivalent to that of the pure cement paste C1(C100) at various ages. Previous studies have also shown that LS and MK have a synergic effect, and LS can interact chemically with aluminate [27,41]. The compressive strength of L3(C80L5M15) blended paste at 3, 7, and 28 days was slightly higher than that of C1(C100) paste. The M7(C82.5L10M7.5) blended paste showed a similar strength to C1(C100) in the early stage (i.e., 3, 7 days). The difference of strengths between the two specimens was less than 2.7 MPa. At 28 days, the compressive strength of M7(C82.5L10M7.5) was 21.5% lower than that of C1(C100). This may be due to the large amount of LS added [42]. Observing Figure 6a,b, it can be seen that the amount of replacement alternative materials was greater in the ternary- and quaternary-blended pastes L4(C65L5F30), L5(C50L5M15F30), M8(C62.5M7.5F30), and M9(C52.5L10M7.5F30), which had a significant decrease at each age. There may be two reasons for that, the main reason being that the cement content was too small, and the other reason was that the decrease was caused by too much FA substitution. Previous studies have shown that the addition of FA can reduce the compressive strength of concrete [43]. As shown in Figure 6c, compared with the C1(C100) paste, the compressive strength of the F10(C85F15) blended paste with 15% FA added at 3, 7, and 28 days was approximately 6.6, 5.7, and 13.5 MPa lower than that of the C1(C100) paste, respectively. In addition, compared with the F10(C85F15) blended paste, F11(C75L10F15) with 10% LS was also slightly reduced. It is worth noting that the F12(C70M15F15) blended paste exhibited a compressive strength equivalent to that of the C1(C100) paste at all ages. Previously, Sujjavanich et al. [44] reported similar experimental results, indicating that when the ration of MK to FA is 1, a strong chemical interaction occurs between the aluminum phases in the blended paste, and this effect produces a synergistic effect that can contribute to compressive strength development. The compressive strength of quaternary-blended paste F13(C60L10M15F15) was slightly lower than that of the F12(C70M15F15) blended paste, but the 28-day strength was still higher than that of the binary-blended paste F10(C85F15) and ternary=blended paste F11(C75L10F15).

### 3.4. Ultrasonic Pulse Velocity

**Figure 7:**
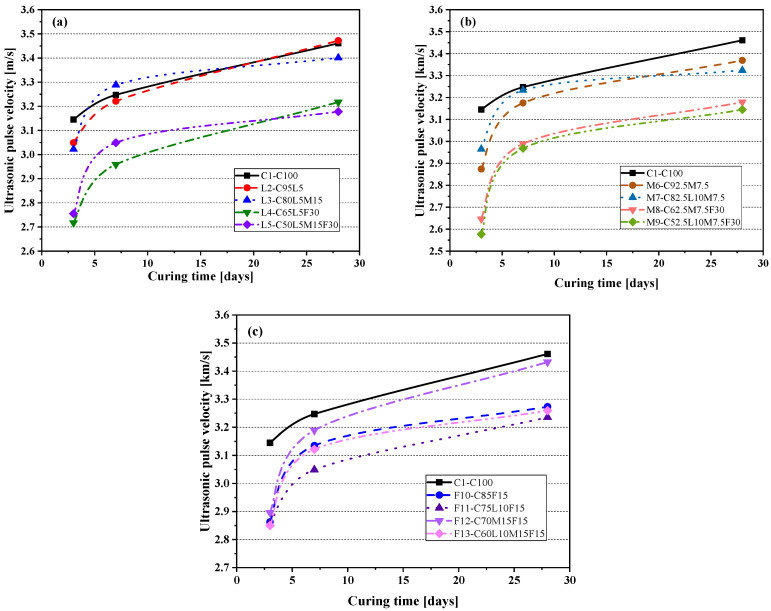
Ultrasonic pulse velocity: (**a**) control specimen and LS system; (**b**) control specimen and MK system; (**c**) control specimen and FA system.

Ultrasonic pulse velocity (UPV) is a commonly used non-destructive testing method that evaluates the long-term sustainability of concrete structures (bridges, buildings, etc.). The UPV is determined by detecting the time required for the longitudinal ultrasonic pulse passing through the paste. Figure 7 shows the test results of the ultrasonic pulse velocity of the paste in this article. The pure cement paste’s ultrasonic pulse velocities at 3, 7, and 28 days were 3.145, 3.247, and 3.461 km/s, respectively. In the early hydration stage (3 days), the UPV of the paste with alternative materials was lower than the control paste, C1(C100, 3.145 km/s). With the increase in the amount of replacement alternative materials, the UPV decreased, but as expected, as the age increased, the UPV gradually increased.

As shown in Figure 7a, compared with the pure cement paste C1(C100), the ultrasonic pulse speeds of the blended paste L4(C65L5F30) and L5(C50L5M15F30) were significantly reduced. This may be caused by too little cement content. The L2(C95L5) blended paste with only 5% LS added had no significant change compared with the C1(C100) paste. The UPV of the L3(C80L5M15) blended paste increased very rapidly in the early stage and exceeded that of pure cement paste at 7 days, but with the increase in the curing age, the value of the UPV at 28 days was slightly lower than that of the C1(C100) paste. The MK system blended paste is shown in Figure 7b. The M8(C62.5M7.5F30) blended paste and the quaternary-blended paste M9(C52.5L10M7.5F30) had a lower UPV value because of the lower cement content. In the early stage (3, 7 days), the UPV value of the M7(C82.5L10M7.5) blended paste was higher than the M6(C92.5M7.5) blended paste, but when cured to 28 days, the M7(C82.5L10M7.5) blended paste was lower than the M6(C92.5M7.5) blended paste. Observing Figure 7c, it can be seen that the UPV values of the FA system blended paste at 3, 7, and 28 days were lower than pure cement paste. The UPV value of the F10(C85F15) blended paste with 15% FA decreased significantly. In summary, adding 5% of LS does not significantly affect the UPV, but when the amount of replacement LS increases by 10% (M7(C82.5L10M7.5), F11(C75L10F15)), the UPV value may decrease slightly because of the decrease in cement content. When MK is added at 7.5%, the UPV of the paste may decrease slightly; when the replacement amount reaches 15%, the UPV of the paste in the early stage (7 days) can increase rapidly. The addition of FA can significantly reduce the UPV of the blended paste, similar to previous studies [45].

### 3.5. Surface Resistivity

**Figure 8:**
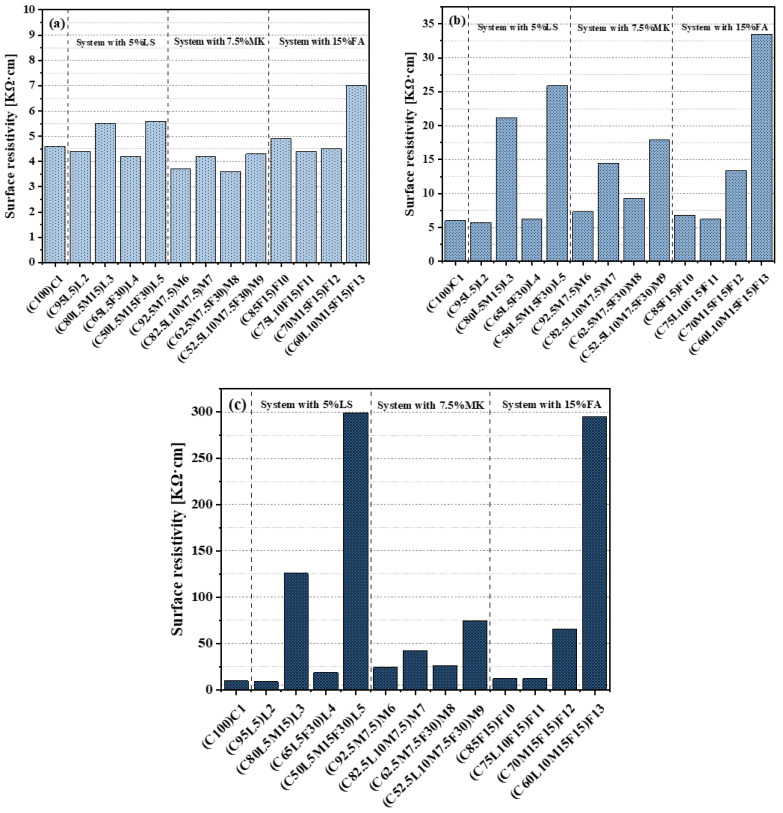
Surface resistivity: (**a**) Surface resistivity at 3 days; (**b**) surface resistivity at 7 days; (**c**) surface resistivity at 28 days.

Figure 8a–c shows the surface resistivity test results of the paste in this paper at 3, 7, and 28 days, respectively. The surface resistivity values of the paste at 3, 7, and 28 days were 3.6–7, 5.7–33.5, and 8.7–299 KΩ·cm, respectively. It was found that as the age increased, the differences between the surface resistivity values of the paste became increasingly significant. Observing Figure 8a, it can be found that as the amount of replacement alternative materials increased, the surface resistivity value did not change significantly. Only the surface resistivity value of the L3(C80L5M15), L5(C50L5M15F30), and F13(C60L10M15F15) blended pastes exceeded 5 KΩ·cm; the highest value was the quaternary-blended paste F13(C60L10M15F15) (surface resistivity = 7 KΩ·cm). As shown in Figure 8b, compared with 3 days (shown in Figure 8a), the surface resistivity of all the blended pastes increased at 7 days. Among them, ternary-blended pastes (such as L3, M7, and F12) and quaternary-blended pastes had a significant increase, but the increase in other blended pastes was not substantial. The 28-day-old paste surface resistivity is shown in Figure 8c; it was found that the difference between the surface resistivity values of the paste became very significant. In particular, the L5(C50L5M15F30) and F13(C60L10M15F15) blended pastes had a very significant increase, with surface resistivity values of 299 and 295 KΩ·cm, respectively. The ternary=blended pastes L3(C80L5M15) and F12(C70M15F15) and the quaternary-blended paste M9(C52.5L10M7.5F30) also increased significantly. In summary, the surface resistivity values of the pastes were mainly affected by the LC^3^ system. The blended pastes with a significant increase in surface resistivity values at 3 and 7 days all contained the LC^3^ system (except F12(C70M15F15)); this may be because the pore structure of the LC^3^ system became denser compared with pure cement pastes in the early days [46]. Previous studies have shown that a 1:2 replacement ratio of LS and MK has the best synergy. As the ratio of LS and MK approaches 1:2, the pore structure of the blended paste improves [47]. Therefore, only the L5(C50L5M15F30) and F13(C60L10M15F15) blended pastes had a very significant increase in surface resistivity at 28 days. Moreover, comparing L3(C80L5M15) and L5(C50L5M15F30), it was found that as the maintenance age increased, the effect of FA became increasingly significant.

### 3.6. XRD

**Figure 9:**
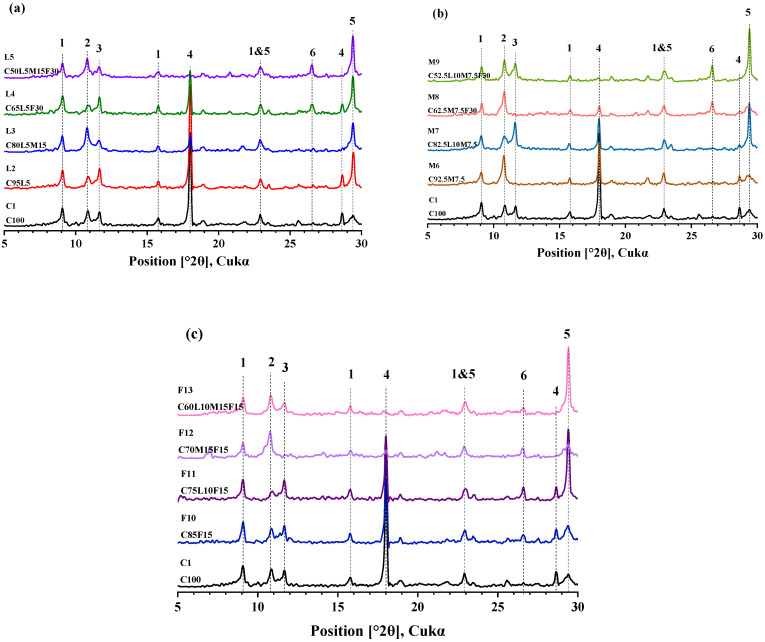
XRD analysis: (**a**) the control specimen and LS system, (**b**) the control specimen and MK system, and (**c**) the control specimen and FA system (1 = ettringite; 2 = hemicarboaluminate; 3 = monocarboaluminate; 4 = portlandite; 5 = calcium carbonate; 6 = quartz).

XRD is usually used to analyze and characterize the mineralogical composition of paste [48]. The 28 day XRD spectra of the three systems in this paper are shown in Figure 9. In the blended paste of the LS system, compared with the pure cement paste, C1(C100), adding 5% LS of the L2(C95L5) blended paste, the peak of monocarboaluminate (Mc) (2θ = 11.6) increased, which indicated that the LS reaction promoted the conversion of hemicarboaluminate (Hc) to Mc [47]; this phenomenon was also found in M7(C82.5L10M7.5), F10(C52.5L10M7.5F30), and F11(C75L10F15). Mc is more stable than Hc in thermodynamics, but it was found that after adding MK, the peak of Hc (2θ = 10.5) became more obvious (L3(C80L5M15), L5(C50L5M15F30) in Figure 9a; F12(C70M15F15), F13(C60L10M15F15) in Figure 9c). This showed that the increase in aluminate in the blended paste was more conducive to Hc formation, which was also consistent with Lin’s previous report [49]. In addition, previous studies have shown that when LS is added to the paste, the conversion of ettringite (AFt) to monosulfate can be inhibited [42]. When calcium carbonate was present, the aluminate in the blended paste preferentially reacted with calcium carbonate to form Mc. For example, in the blended pastes of M7(C82.5L10M7.5) and M9(C52.5L10M7.5F30) in Figure 9b, when the peak of calcium carbonate (2θ = 29.5) existed, there was an obvious Mc peak. When there was no obvious calcium carbonate peak (M6(C92.5M7.5) and M8(C62.5M7.5F30)) in the blended pastes in Figure 9b, the AFt peak (2θ = 9.1) showed signs of weakening. Figure 9 shows the calcium hydroxide (CH) peak produced by cement hydration at 2θ = 18. It was found that as the addition of MK and FA increased, the CH peak gradually weakened, and the CH peaks of the quaternary-blended pastes L5(C50L5M15F30), M9(C52.5L10M7.5F30), and F13(C60L10M15F15) disappeared completely. The presence of CH can adversely affect the durability of the blended paste [50], so the addition of MK and FA with the pozzolanic reaction was also helpful for durability. This was also consistent with the results of the surface resistivity study above (Section 3.5).

### 3.7. ATR-FTIR

**Figure 10:**
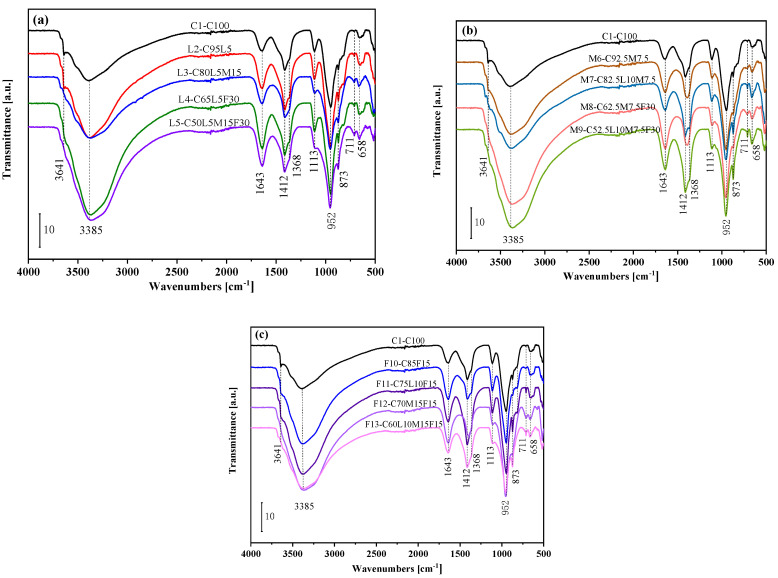
FTIR spectra analysis: (**a**) the control specimen and LS system; (**b**) the control specimen and MK system; (**c**) the control specimen and FA system.

Figure 10 shows the FTIR spectra of the pastes for 28 days. Figure 10a–c represents the blended pastes of the LS system, the MK system, and the FA system, respectively. As shown in the figure, the absorption peak at 3641 cm^−1^ was caused by the stretching vibration of the O–H bond of CH [51]. It was seen that with the increase in the amount of the substitute material, the absorption peak of CH gradually weakened, which was consistent with the results observed by XRD. The absorption bands at 3385 and 1643 cm^−1^ in Figure 10 were caused by the O–H tensile vibration and bending vibration of H_2_O, respectively [52].

The absorption band at 1368–1412 cm^−1^ was caused by the asymmetric stretching vibration of CO_3_^2−^ [53]. It is worth noting that part of the blended paste had a weaker absorption peak at 1368 cm^−1^, which the presence of Mc may cause. The sharp absorption peaks at 873 and 711 cm^−1^ were caused by the out-of-plane bending vibration of CO_3_^2−^ [49]. The SO_4_^2−^ asymmetric stretching vibration caused the absorption peak to appear at 1113 cm^−1^ [49]. It can be seen that the absorption peaks of the L3(C80L5M15) and L5(C50L5M15F30) blended pastes in Figure 10a and the F12(C70M15F15) and F13(C60L10M15F15) blended pastes in Figure 10c were significantly weakened; and in Figure 10b, the absorption peak of the blended paste of the MK system was also weakened. This may be due to the reaction of aluminate in MK with sulfate ions (SO_4_^2−^) to form AFt. The last absorption peaks at 952 and 658 cm^−1^ were caused by the asymmetric stretching vibration and bending vibration of the Si–O bond, respectively [49,54]. In Figure 10a, the blended paste absorption peak with 5% LS added at 952 cm^−1^ had a significant increase, which may be because the nucleation of LS provided nucleation sites for CSH [55], resulting in more CSH. In Figure 10b, the M6(C92.5M7.5) blended paste’s absorption peak at 952 cm^−1^ also increased significantly. This may be due to the MK pozzolanic effect, which reacted with CH to generate more CSH. At 3641cm^−1^ in Figure 10b, a decrease in the absorption peak of CH was also observed accordingly.

### 3.8. CO_2_ Emissions

**Figure 11:**
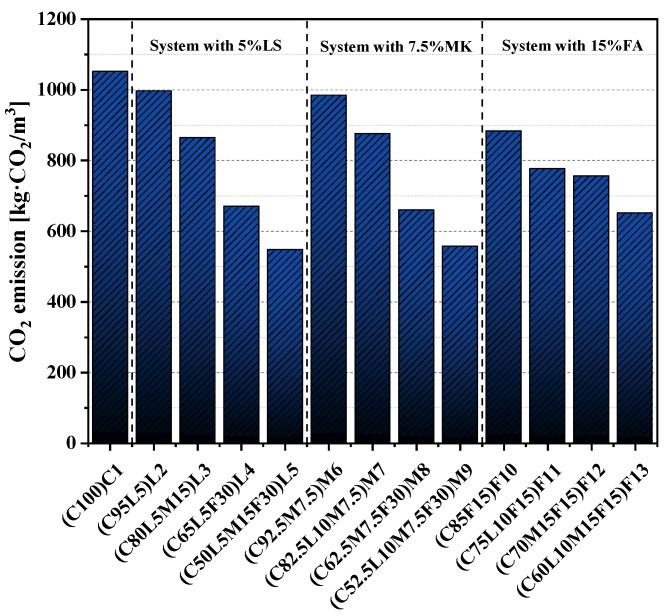
CO_2_ emissions of unit volume of paste.

Table 4 lists the CO_2_ emission factors per unit of OPC and the three alternative materials used in this paper’s experimental study [56]. The unit emission of CO_2_ studied in this paper did not consider material transportation and other treatment processes and was only determined based on the CO_2_ emission factor and the water–cement ratio of raw materials. Based on the unit CO_2_ emissions and mixtures of specimens, the CO_2_ emissions of unit volume specimens were determined. Figure 11 shows the CO_2_ emissions per unit volume of paste. The CO_2_ emission of pure cement paste was 1052.04 kg·CO_2_/m^3^, which was the highest among the pastes. In the three hybrid systems, with the increase in the replacement of alternative materials, the CO_2_ emissions all showed a gradual decrease. The CO_2_ emissions of the three quaternary-blended pastes L5(C50L5M15F30), M9(C52.5L10M7.5F30), and F13(C60L10M15F15) in this study were 548.09, 557.66, and 651.5 kg·CO_2_/m^3^, respectively, compared with pure cement paste. This was a reduction of 38.07 to 47.9%.

## 4. Discussions

### 4.1. Correlation Analysis

**Figure 12:**
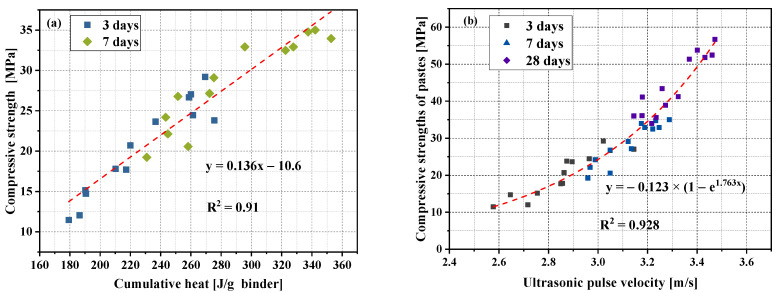
Relationships among various test results. (**a**) Relationships between cumulative hydration heat and compressive strength at curing ages of 3 days; (**b**) relationships between compressive strength and UPV at curing ages of 3, 7, and 28 days.

Li et al. [33] used Pearson’s correlation coefficient to study the correlation between the cumulative heat of hydration and compressive strength and reported a strong correlation between hydration and compressive strength. In this article, the hydration heat release and compressive strength at 3 and 7 days were experimentally studied, and this was also confirmed. Figure 12a shows the relationship between the cumulative heat of hydration and compressive strength. There was a good correlation between the cumulative amount of hydration heat and the compressive strength. Pearson’s correlation coefficient between strength and hydration heat was 0.95. The R^2^ fitted by the linear equation regression model was 0.91.

As is known, many factors affect the ultrasonic velocity, such as cement type, water–cement ratio, alternative materials, and environmental temperature [57]. In this study, when other influencing factors, such as cement material and the water–cement ratio, are fixed, the amount of replacement alternative materials is the main factor affecting the ultrasonic velocity. Similarly, the amount of replacement alternative materials can have a great impact on the compressive strength. Figure 12b shows the relationship between the compressive strength of the paste in this paper at 3, 7, and 28 days and the ultrasonic pulse velocity. There was a very significant exponential relationship between the strength and UPV. Pearson’s correlation coefficient between strength and UPV was 0.96. The R^2^ fitted by the exponential equation model was 0.928. Many previous studies have confirmed this [58,59].

The paste maturation rate means the average reaction degree of binders. For the plain paste, the hydration degree of Portland cement is the maturation rate, while for blended paste, the average reaction degree of cement and mineral admixture is the maturation rate. UPV is mainly dependent on the contents of solid hydration products, while electrical resistivity depends on various factors, such as degree of saturation, pore size distribution, and ion concentration of pore solution.

For the plain paste, the results of both UPV and electrical resistivity were used to measure the maturation rate. For the blended paste, from 3 days to 28 days, the increment content of UPV was comparable with that of strength, while the increment content of electrical resistivity was much higher than that of strength because of the pore size refinement from the mineral admixtures reaction. Hence for blended paste, compared with electrical resistivity, UPV was a more suitable indicator to measure the maturation rate.

### 4.2. Sustainability Analysis

Because of the low unit carbon dioxide emission factor of SCMs, SCMs reduce carbon dioxide emissions when replacing cement. Still, the addition of SCMs also affects the performance of concrete. Therefore, to further evaluate the pros and cons of alternative materials in terms of sustainability, the CO_2_ emissions were normalized according to the 28-day compressive strength of the blended pastes, as shown in Equation (1):

(1) $$E_{f_{c}}=\frac{CO_{2-m^{3}}}{f_{c-28}}$$

where $E_{f_{c}}$ is strength-normalized CO_2_ emission (kg·CO_2_/MPa), $CO_{2-m^{3}}$ is the CO_2_ emissions per cubic meter of blended pastes (shown in Figure 11), and $f_{c-28}$ is the value of the compressive strength of the blended pastes sample at 28 days.

**Figure 13:**
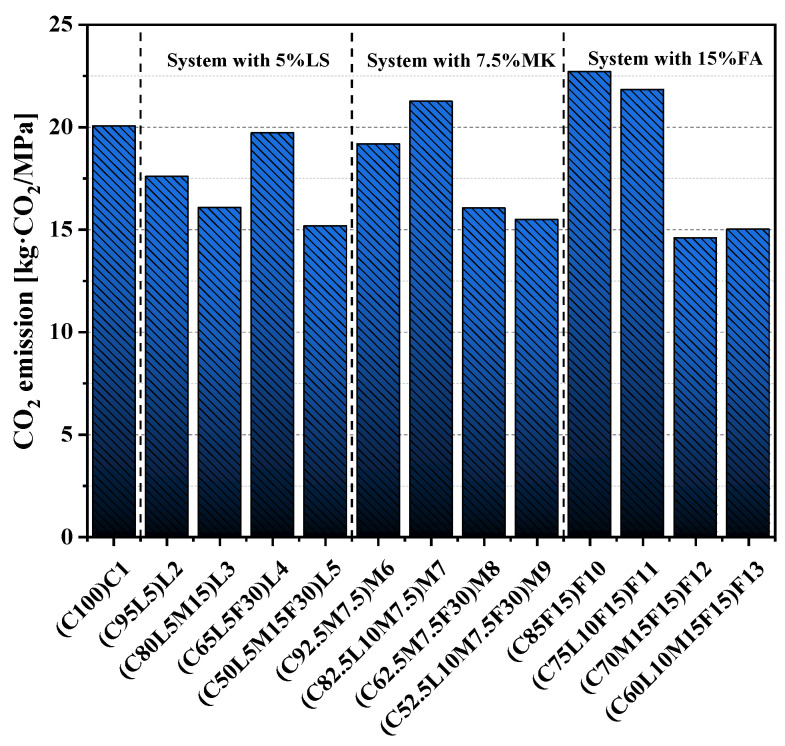
CO_2_ emissions per unit of compressive strength.

The results of strength-normalized CO_2_ emissions are shown in Figure 13. The F10(C85F15) blended paste had the highest normalized CO_2_ emissions of all pastes, which was 22.72 kg·CO_2_/MPa. This may be because the addition of 15% FA significantly reduced the blended paste’s compressive strength. In addition, the normalized CO_2_ emissions of M7(C82.5L10M7.5) and F11(C75L10F15) exceeded C1(C100). The F12(C70M15F15) blended paste had the lowest CO_2_ emission per unit intensity, 14.8 kg·CO_2_/MPa. This showed that when MK and FA were added simultaneously in a ratio of 1:1, they had a significant effect on reducing CO_2_ emissions while ensuring compressive strength. For quaternary-blended paste, the CO_2_ emissions per unit strength was far less than that of the pure cement paste, respectively, 15.47 kg·CO_2_/MPa (L5(C50L5M15F30)), 15.63 kg·CO_2_/MPa (M9(C52.5L10M7.5F30)), and 15.25 kg·CO_2_/MPa (F13(C60L10M15F15)). Therefore, the quaternary-blended pastes had an obvious positive role in sustainable development.

## 5. Conclusions

This article used three supplementary cementitious materials, limestone powder (0–10%), metakaolin (0–15%), and fly ash (0–30%), and binary-, ternary-, and quaternary-blended pastes were prepared. According to the test results, the following conclusions can be drawn:

The influence of metakaolin on workability was negative, but the fly ash increased the workability of the blended paste owing to the ball-bearing effect.

(1) Fly ash can effectively reduce the accumulated heat of hydration of the blended paste. The cumulative heat and compressive strength also showed an excellent linear relationship (Pearson’s correlation coefficient of 0.95).
(2) The heat flow curve of the blended pastes L3, L5, and F13 showed a reaction peak caused by carboaluminate (Hc and Mc) production at about 60 h, which rapidly increases the accumulated heat release, also at 60 h.
(3) The nucleation effect of limestone powder is helpful for improvement of early-age strength. LS and MK have a synergic effect for strength development. Too much FA substitution lowers compressive strength.
(4) The addition of alternative materials was the main factor affecting the ultrasonic pulse velocity. In addition, the ultrasonic pulse velocity had a strong correlation with the compressive strength; Pearson’s correlation coefficient was 0.96.
(5) The surface resistivity test results found that the quaternary-blended paste had a very significant positive effect on the durability of concrete. At 28 days, the quaternary-blended pastes L5 and F13 reached 299 and 295 KΩ·cm, respectively.
(6) The combined analysis of XRD and FTIR confirmed that when limestone powder is added, aluminate can preferentially consume calcium carbonate to form Hc and Mc. Furthermore, the presence of aluminate was more conducive to Hc’s formation.
(7) After standardizing the compressive strength and CO_2_ emissions, we found that the normalized CO_2_ emissions of the quaternary-blended pastes were less than that of the pure cement paste. Therefore, the quaternary-blended pastes had an obvious positive role in sustainable development.

## Figures and Tables

**Table 1:** Chemical compositions of materials in weight % (OPC: ordinary Portland cement; LS: limestone powder; MK: metakaolin; FA: fly ash).

Chemical Compositions	OPC	LS	MK	FA
SiO_2_	20.6	-	52.1	50.3
Al_2_O_3_	4.74	0.19	45.2	20.52
Fe_2_O_3_	2.66	-	0.253	7.87
CaO	62.3	59.14	0.302	9.97
K_2_O	0.957	-	0.08	1.53
MgO	2.59	0.41	-	3.13
Na_2_O	-	-	-	0.92
TiO_2_	0.291	-	0.514	0.87
SO_3_	2.51	-	-	0.301
LOI ^a^	2.57	39.52	0.989	3.925

^a^ Loss on ignition.

**Table 2:** Physical properties of materials.

	OPC	LS	MK	FA
d50 (μm)	17.8	11.6	3.84	30.6
Specific gravity	3.14	2.74	2.50	2.35

**Table 3:** Mix proportions of specimens.

Mixed No.	OPC (wt.%)	LS (wt.%)	MK (wt.%)	FA (wt.%)	OPC (kg/m^3^)	LS (kg/m^3^)	MK (kg/m^3^)	FA (kg/m^3^)
Control specimen								
C1(C100)	100	0	0	0	1223.3	-	-	-
System with 5% LS								
L2(C95L5)	95	5	0	0	1158.77	60.99	-	-
L3(C80L5M15)	80	5	15	0	961.29	60.08	180.24	-
L4(C65L5F30)	65	5	0	30	762.68	58.67	-	352.01
L5(C50L5M15F30)	50	5	15	30	578.28	57.83	173.48	346.97
System with 7.5% MK								
M6(C92.5M7.5)	92.5	0	7.5	0	1123.05	-	91.06	-
M7(C82.5L10M7.5)	82.5	10	7.5	0	995.89	120.72	90.54	-
M8(C62.5M7.5F30)	62.5	0	7.5	30	730.08	-	87.61	350.44
M9(C52.5L10M7.5F30)	52.5	10	7.5	30	609.88	116.17	87.13	348.5
System with 15% FA								
F10(C85F15)	85	0	0	15	1019.59	-	-	179.93
F11(C75L10F15)	75	10	0	15	894.54	119.27	-	178.91
F12(C70M15F15)	70	0	15	15	827.37	-	177.29	177.29
F13(C60L10M15F15)	60	10	15	15	705.22	117.54	176.3	176.3

**Table 4:** CO_2_ emission factor of materials [56].

Raw Material Limestone	OPC	LS	MK	FA
CO_2_ emission factor (kg·CO_2_/kg)	0.86	0.008	0.21	0.04

## Data Availability

The data presented in this study are available from the corresponding author upon reasonable request.
